# Characterization and experimental verification of the rotating synthetic aperture optical imaging system

**DOI:** 10.1038/s41598-023-44382-2

**Published:** 2023-10-09

**Authors:** Yu Sun, Xiyang Zhi, Lei Zhang, Shikai Jiang, Tianjun Shi, Nan Wang, Jinnan Gong

**Affiliations:** 1 Research Center for Space Optical Engineering, Harbin Institute of Technology, Harbin, 150001 China; 2 Beijing Institute of Tracking and Telecommunications Technology, Beijing, 100094 China; 3 Foreign Studies College, Northeastern University, Shenyang, 110001 China

**Keywords:** Optical techniques, Imaging and sensing

## Abstract

The rotating synthetic aperture (RSA) optical imaging system employs a rectangular primary mirror for detection. During the imaging process, the primary mirror rotates around the center to achieve the aperture equivalent to the long side of the rectangle at different rotation angles. As a result, the system’s point spread function changes over time, causing periodic time-varying characteristics in the acquired images’ resolution. Moreover, due to the rectangular primary mirror, the images obtained by the RSA system are spatially asymmetric, with a lower resolution in the short side’s direction than in the long side’s direction. Hence, image processing techniques are necessary to enhance the image quality. To provide reference for the study of image quality improvement methods, we first characterize the imaging quality degradation mechanism of the RSA system and the time–space evolution law of the imaging process. We then establish an imaging experiment platform to simulate the dynamic imaging process of the RSA system. We quantify the RSA system’s impact on image degradation using objective indexes. Subsequently, by comparing the imaging experiment results with theoretical analysis, we verify the spatially asymmetric and temporally periodic imaging characteristics of the RSA system. Lastly, we introduce image super-resolution experiments to assess the limitations of directly applying generic deep learning-based single image super-resolution methods to the images captured by the RSA system, thereby revealing the challenges involved in improving image quality for the RSA system.

## Introduction

High orbit remote sensing satellites rely mainly on large aperture primary mirrors to achieve high spatial resolution and image quality^1–4^. However, the traditional refraction/reflection optical system with a large aperture monomer primary mirror as the core is limited by the rocket’s carrying capacity, as well as its own system complexity, volume, weight, and cost, making it challenging to meet the requirements of lightweight and low-cost applications^5–7^. To address these limitations, the rotating synthetic aperture (RSA) imaging technology was developed, which employs a rectangular primary mirror with a large aspect ratio. During the imaging process, the primary mirror rotates around the center to achieve the aperture equivalent to the long side of the rectangle at different rotation angles. The RSA system is lightweight, easy to manufacture, and one of the most promising technical approaches for optical remote sensing in stationary orbit^8^.

Back in the 1980s, the rotating slit aperture telescope, a 1D analog of the RSA system, was proven to be a promising alternative to the classical telescope for achieving high-resolution astronomical imagery^9,10^. The concept of the RSA system emerged from the realization that a rotating rectangular aperture can achieve a larger effective aperture while maintaining a smaller mass. Consequently, this system gradually found its application in high-resolution remote sensing missions. In 1995, a collaborative research project on rotating synthetic aperture was initiated by Northrop Grumman and Raytheon Company in the United States. The imaging system developed through this collaboration achieved an equivalent aperture of 20 m, all while maintaining the same mass as an 8–9 m monolithic mirror imaging system. This system was successfully deployed in 2015^11^. Subsequently, Nir et al. performed simulations to compare images acquired using telescopes with elongated and circular pupils, both having identical aperture areas^12^. According to their simulations, the elongated-pupil telescope achieves greater contrast at lower separation between a faint companion and a bright star.

Although the RSA system offers several advantages, such as a lightweight primary mirror, elimination of the need for splicing, and the absence of real-time surface shape maintenance in orbit, there are certain drawbacks in its sequential imaging process. Specifically, the rotation of the primary mirror introduces periodic changes in image quality, and the short side direction of the primary mirror yields lower image quality compared to the long side direction^13^. The point spread function (PSF) of the primary mirror changes over time and is coupled with satellite platform vibrations, primary mirror rotation errors, detector sampling, and imaging process noise, leading to the RSA system’s unique degradation mechanism that is both time-periodic and spatially asymmetric^14^. Therefore, image processing methods must be employed to restore and improve the system’s imaging quality. Several relevant studies have proposed methods to improve image quality specifically for this system. Zackay et al. introduced a non-blind restoration method for star detection using matched filters, but the fusion result is unclear^15,16^. Zhou et al. proposed a method utilizing Hyper-Laplacian and sparse priors^17^. Lv et al. proposed a full-aperture image synthesis method based on Fourier spectrum restoration^18^. While these methods demonstrate satisfactory synthesis effects for sequence images with significant noise, they fail to adequately address the challenges of image registration arising from satellite platform vibrations during on-orbit applications. To address this issue, Zhi et al. presented an image registration and restoration method based on the theory of mutual information^8^. However, the aforementioned methods all fall under the category of traditional image quality enhancement methods that require multiple input frames. With the advancement of deep learning technology, neural networks are gradually being utilized for enhancing image quality. Deep learning methods, through trainable feature extractors, can adaptively learn the mapping relationship between low-quality and high-quality images, outperforming traditional methods. Consequently, they have become the mainstream approach in recent years. This progress enables the application of single image super-resolution (SR) or restoration methods to the RSA system.

Hence, we first explore the imaging degradation mechanism and imaging characteristics of the RSA system. Next, we conduct semi-physical imaging experiments to simulate the dynamic imaging process of the system. Building upon this foundation, we evaluate several representative deep learning-based image quality enhancement methods using the images obtained from the semi-physical imaging experiments. Considering that the non-circular symmetry of the aperture mainly leads to a significant decrease in resolution along the shorter side of the rectangle, we choose image SR methods such as SRGAN^19^, EDSR^20^, SRMD^21^, FSSR^22^, and Real-ESRGAN^23^ instead of restoration methods. These experiments and analyses contribute to a deeper understanding of the challenges in enhancing image quality for the RSA system, providing a valuable point of reference for further research on image quality improvement methods for this system.

The remainder of this paper is structured as follows. In “Theoretical analysis of imaging characteristics of the RSA system” section provides an analysis of the imaging mechanism of the RSA system. In “Imaging apparatus and experimental scheme” section describes the experimental platform used to simulate the RSA system, as well as the experimental methodology. In “Results and discussion” section, we present and discuss the results of imaging experiments and super-resolution experiments. Finally, in “Conclusion” section, we draw conclusions.

## Theoretical analysis of imaging characteristics of the RSA system

**Figure 1:**
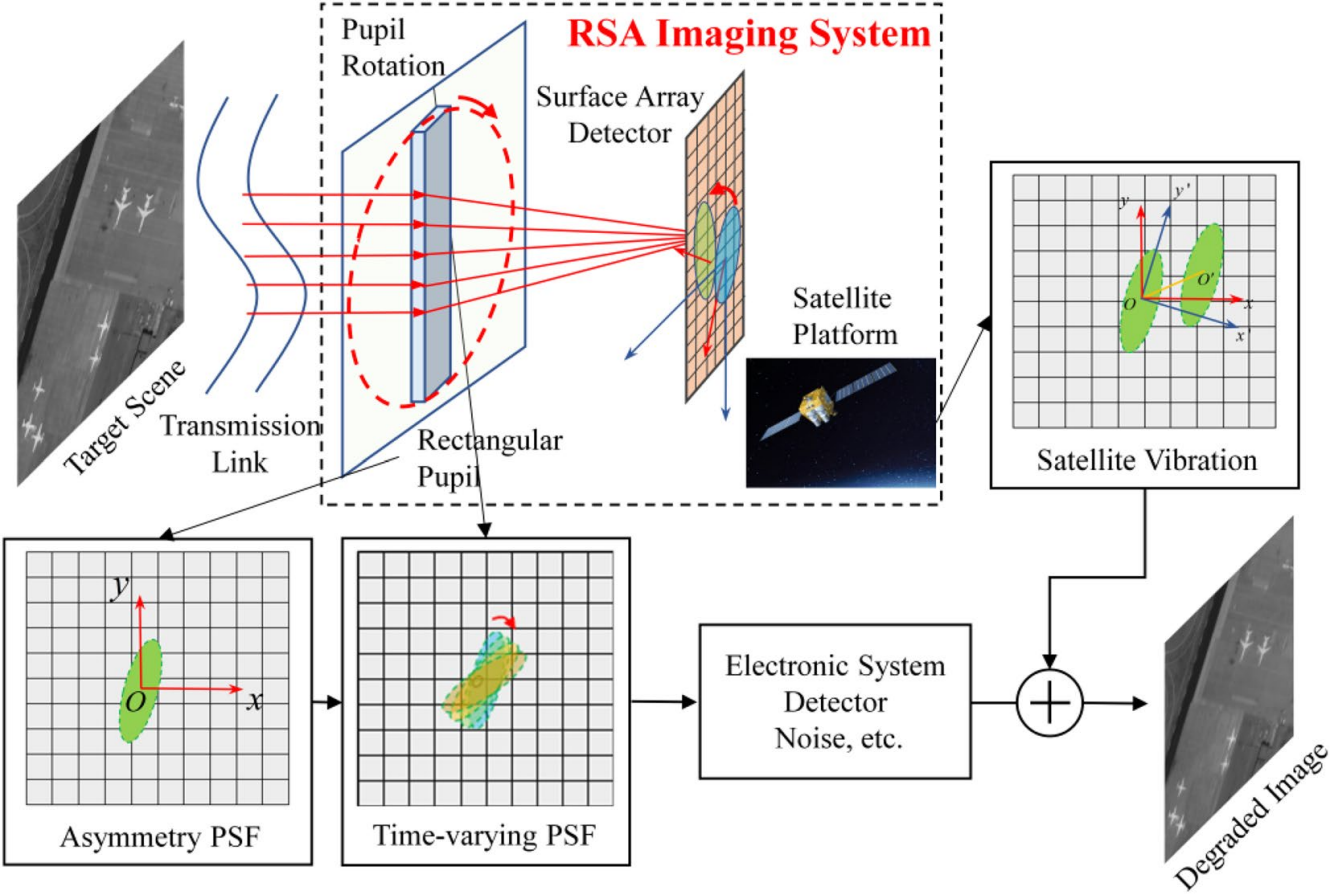
Imaging mode of the rotating synthetic aperture (RSA) system.

Traditional optical remote sensing systems typically employ circular primary mirrors with circular pupils, resulting in circularly symmetric PSFs that exhibit identical degradation characteristics in all directions. In contrast, during imaging, the RSA system rotates a rectangular primary mirror while keeping the two-dimensional array detector fixed relative to the target scene, as shown in Fig. 1. At different times (*T*_0_, *T*_1_,…,*T*_*n*_), the system captures images of the target scene at different rotation angles of the primary mirror. Since the PSF of the rectangular pupil is narrow in the direction of the long side of the rectangle, the image has a higher resolution in the direction of the long side in the process of single imaging. In one rotation period, the system acquires multiple degraded images that preserve the high-resolution information in all directions of the target scene. However, the pixel size limitation of the detector leads to further down-sampling of the high-resolution information in these images during the detector sampling process. The PSF of the rectangular pupil is analyzed below. Specifically, the RSA system’s pupil function is a rectangular function:

1 $$P(\xi ,\eta ) = {\text{rect}}\left( {\frac{\xi }{a}} \right){\text{rect}}\left( {\frac{\eta }{b}} \right) = \left\{ {\begin{array}{*{20}l} {1,} \hfill & {0 \le \xi \le a\;{\text{and}}\;0 \le \eta \le b} \hfill \\ {0,} \hfill & {\text{else }} \hfill \\ \end{array} } \right.$$

During the imaging process of the RSA system, the primary mirror’s rotation results in a periodic change of the pupil function, and the pupil function at time *t* can be expressed as:

2 $$P(\xi ,\eta ,t) = {\text{rect}}\left( {\frac{{\xi \cos (wt + \varphi_{0} ) - \eta \sin (wt + \varphi_{0} )}}{a}} \right){\text{rect}}\left( {\frac{{\xi \sin (wt + \varphi_{0} ) + \eta \cos (wt + \varphi_{0} )}}{b}} \right)$$

where $(\xi ,\eta )$ correspond to the spatial coordinates, *a* and *b* denote the width and height of the primary mirror, *w* represents the rotation angular velocity, and $\varphi_{{0}}$ is the initial phase.

From the aforementioned pupil function, the PSF of the system at time *t* can be determined (i.e., the energy distribution of the primary mirror’s Fraunhofer diffraction pattern):

3 $$\begin{aligned} PSF\left( {x,y,t} \right) & = \left| {e^{{i\pi \frac{{x^{2} + y^{2} }}{\lambda z}}} \iint_{D} P(\xi ,\eta ,t)\exp \left[ { - i2\pi \left( {\frac{x}{\lambda z}\xi + \frac{y}{\lambda z}\eta } \right)} \right]d\xi d\eta } \right|^{2} = |F\{ P(\xi ,\eta ,t)\} |^{2} \\ & { = }ab\;{\text{sinc}}(a(x\cos (wt + \varphi_{{0}} ) - y{\text{sin}}(wt + \varphi_{{0}} ))) \times {\text{sinc}}(b(x\sin (wt + \varphi_{{0}} ) + y{\text{cos}}(wt + \varphi_{{0}} ))) \\ \end{aligned}$$

where *z* represents the imaging distance, $\lambda$ represents the wavelength, and $(x,y)$ represent the spatial coordinates. The operator *F*{} denotes the Fourier transform.

**Figure 2:**
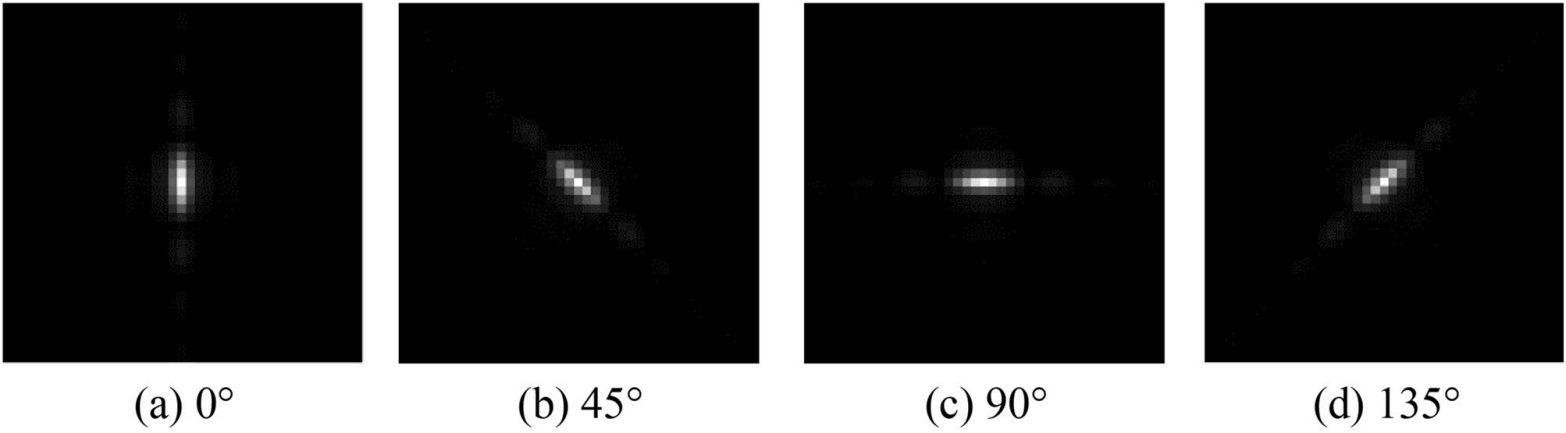
Point spread functions of the RSA system’s primary mirror with different rotation angles.

The PSF of the system’s primary mirror with varying rotation angles is illustrated in Fig. 2. As can be observed, the PSF is roughly elliptical if the secondary diffraction effect is ignored. The shape of the ellipse is determined by the length–width ratio of the rectangular primary mirror, with the direction of the long axis corresponding to the short side.

## Imaging apparatus and experimental scheme

**Figure 3:**
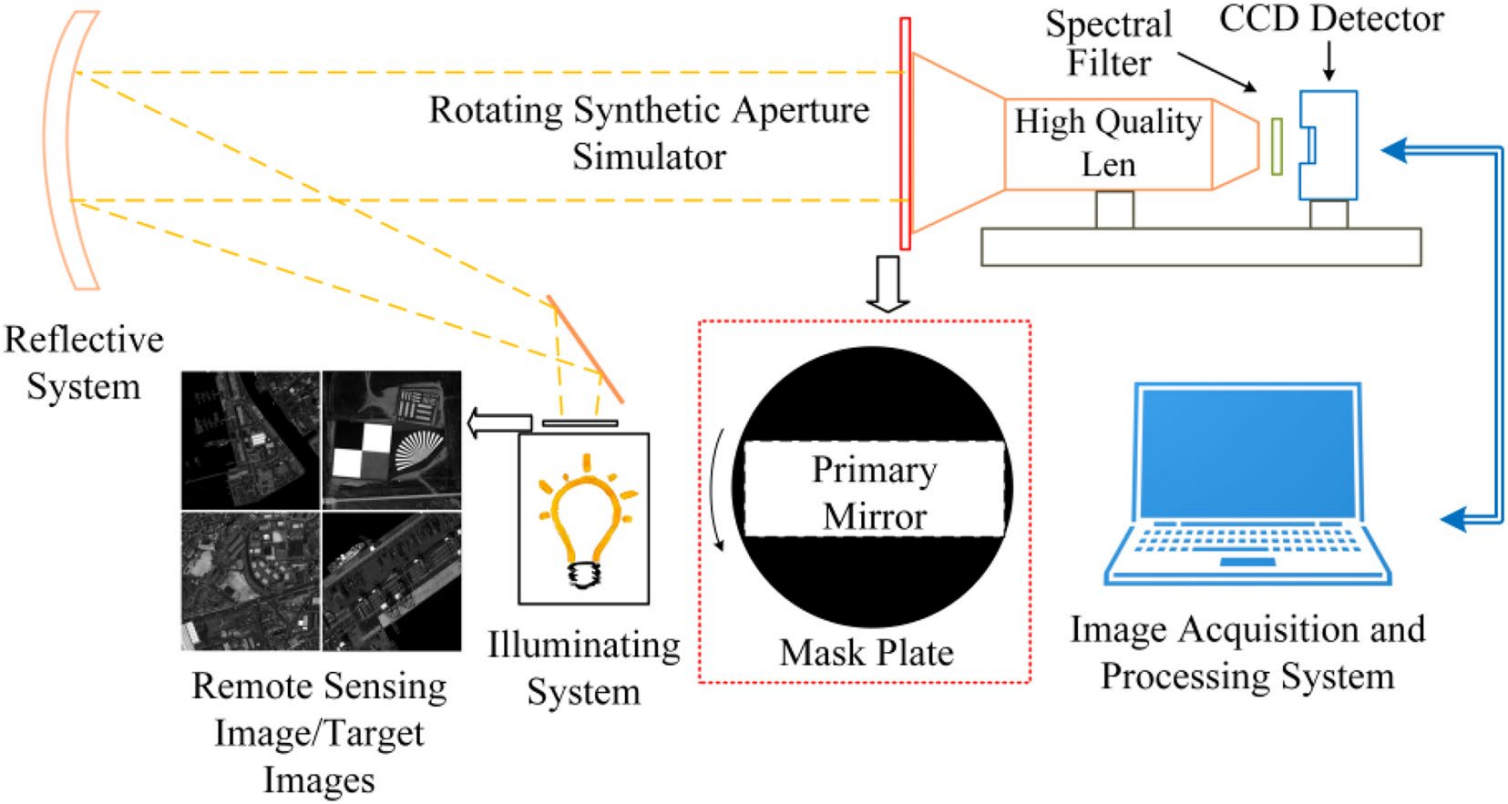
Design scheme of the imaging experiment platform.

**Figure 4:**
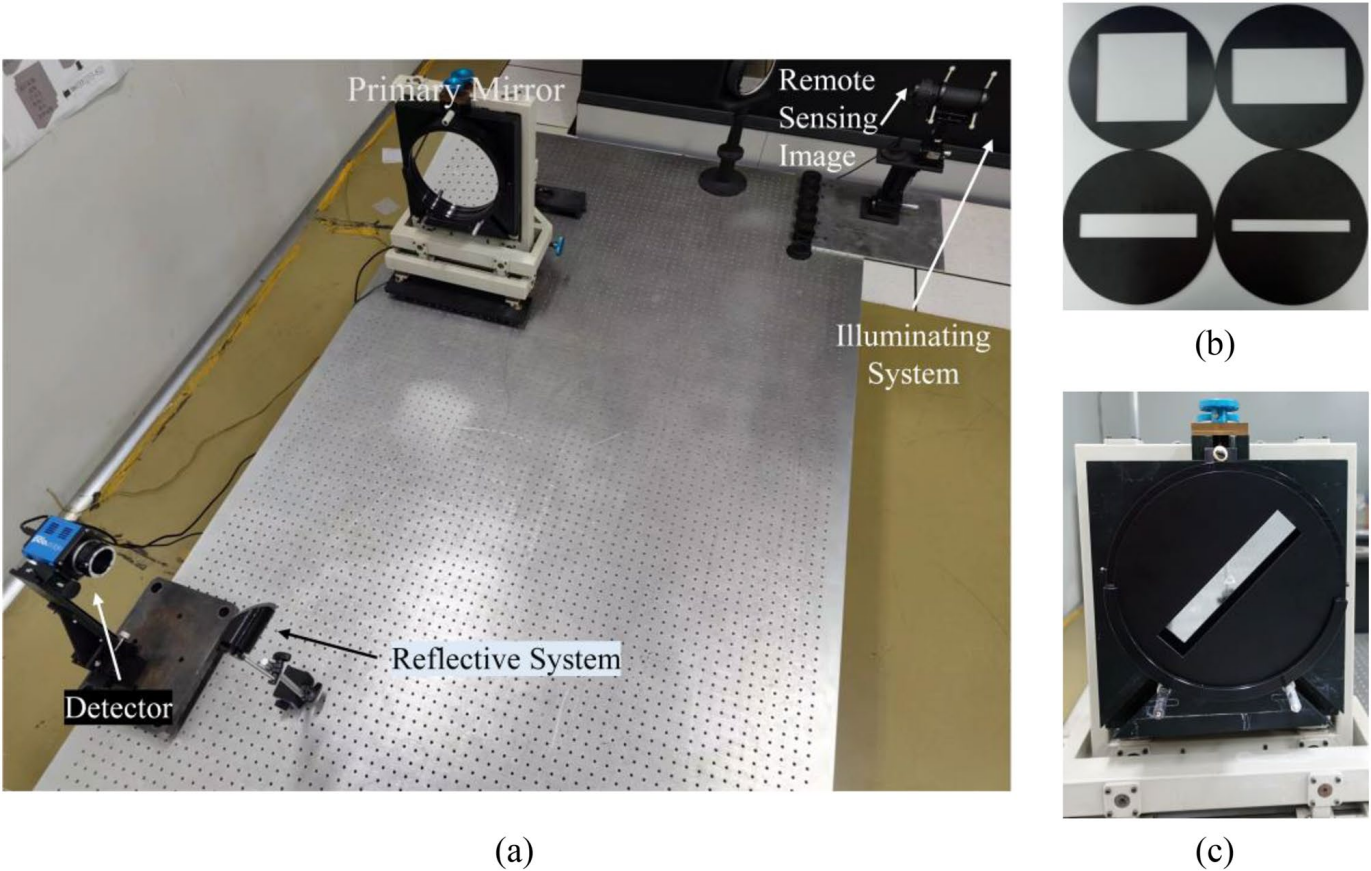
(**a**) The imaging experiment facilities. (**b**) The rectangular pupil optical elements. (**c**) The primary mirror with an element is placed in front.

We develop an experimental platform to simulate the dynamic imaging process of the RSA system, as depicted in Fig. 3. The platform is equipped with a high-quality round primary mirror, a removable and rotary rectangular entry pupil optical component at the front end of the primary mirror, and a spectral filter at the front end of the detector to simulate the effect of different spectral widths. The detector involves sampling, noise effects, and signal conversion. In the experiment, we use sophisticated cartographic equipment to produce high-resolution remote sensing images as target scenes. To simulate the imaging characteristics of the RSA system, we design a rectangular entry pupil optical component and its supporting structure. The rotating motion of the optical component is used to simulate the rotating imaging of the rectangular primary mirror. Figure 4 shows the experimental platform and some experimental components. We first image the target scene using the round primary mirror and then install entrance pupil optical components with different length–width ratios. By rotating them at different angles to image the same target scene, we can obtain a series of low-quality images with high-resolution information in different directions. These images can be combined with the images acquired through the round aperture system to form high-low quality image pairs. The datasets constructed by this method can provide data support for corresponding image quality improvement methods. Furthermore, the PSF of the system can be estimated by using the experimental platform to image star-hole plates, which can provide prior knowledge for corresponding image quality improvement methods.

## Results and discussion

**Figure 5:**
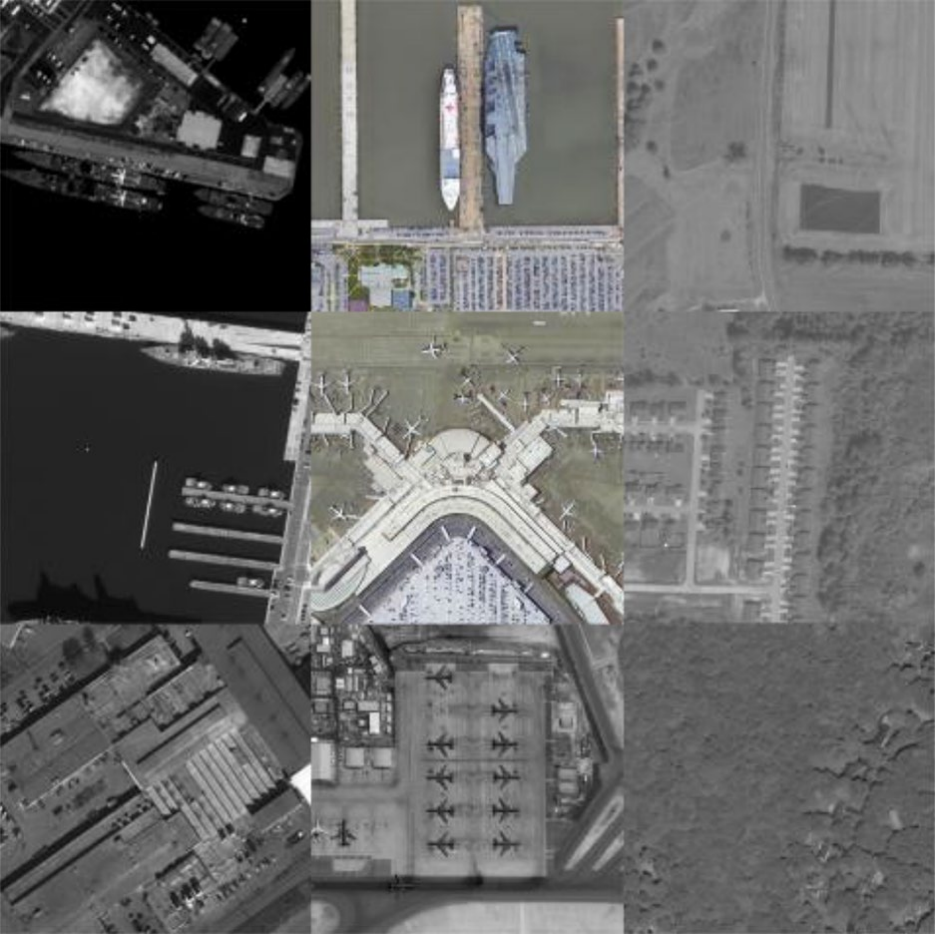
Target scenes.

**Figure 6:**
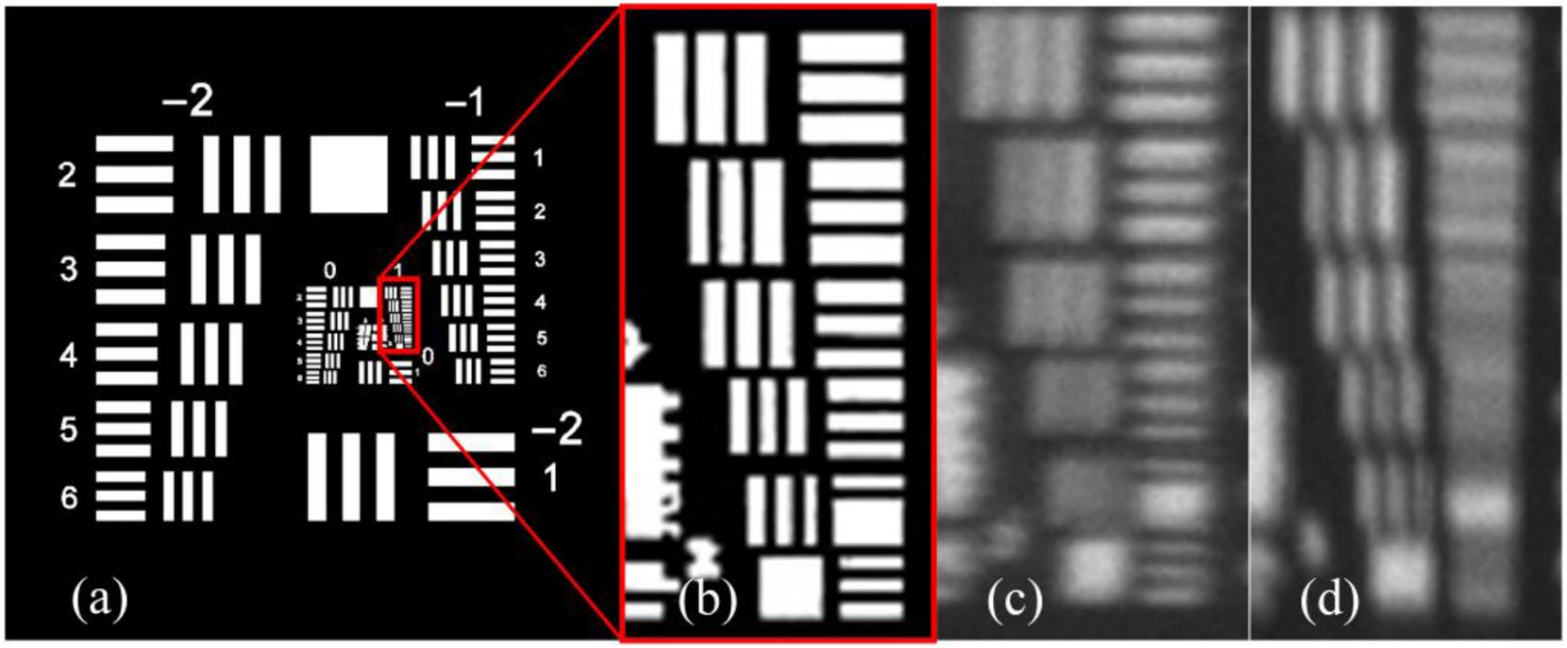
Imaging experiment results when the length–width ratio of the rectangular pupil is 8. (**a**) Original scene. (**b**) Local enlargement image. (**c**) Image taken when the rotation angle of the rectangular pupil is 0. (**d**) Image taken when the rotation angle of the rectangular pupil is 90.

**Figure 7:**
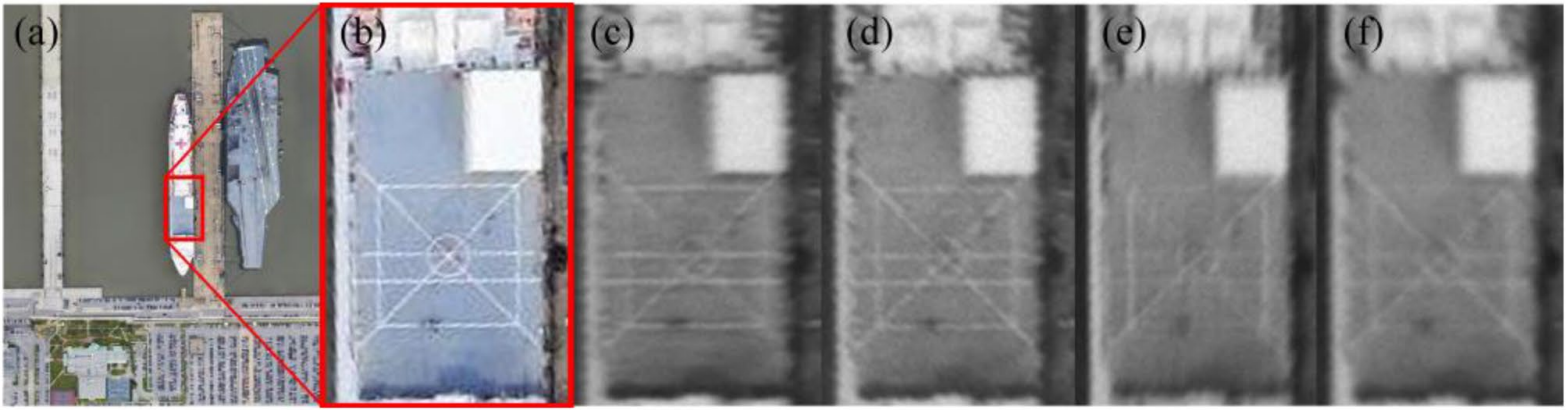
Imaging experiment results when the length–width ratio of the rectangular pupil is 5. The ground resolution of the image is 0.4 m, signifying that every pixel corresponds to an approximate real-world ground distance of 0.4 m. The original remote sensing scene was acquired by the WorldView-3 satellite. (**a**) Original scene. (**b**) Local enlargement image. (**c**) Image taken when the rotation angle of the rectangular pupil is 0. (**d**) Image taken when the rotation angle of the rectangular pupil is 45. (**e**) Image taken when the rotation angle of the rectangular pupil is 90. (**f**) Image taken when the rotation angle of the rectangular pupil is 135.

**Figure 8:**
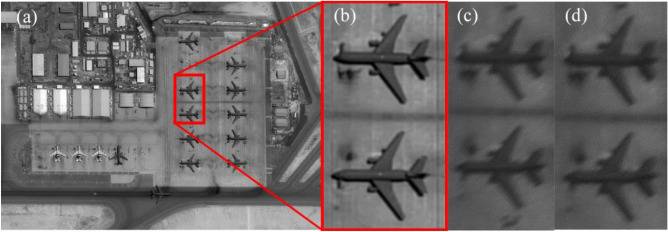
Imaging experiment results when the length–width ratio of the rectangular pupil is 1. The ground resolution of the image is 0.4 m, signifying that every pixel corresponds to an approximate real-world ground distance of 0.4 m. The original remote sensing scene was acquired by the WorldView-3 satellite. (**a**) Original scene. (**b**) Local enlargement image. (**c**) Image taken when the rotation angle of the rectangular pupil is 30. (**d**) Image taken when the rotation angle of the rectangular pupil is 60.

**Figure 9:**
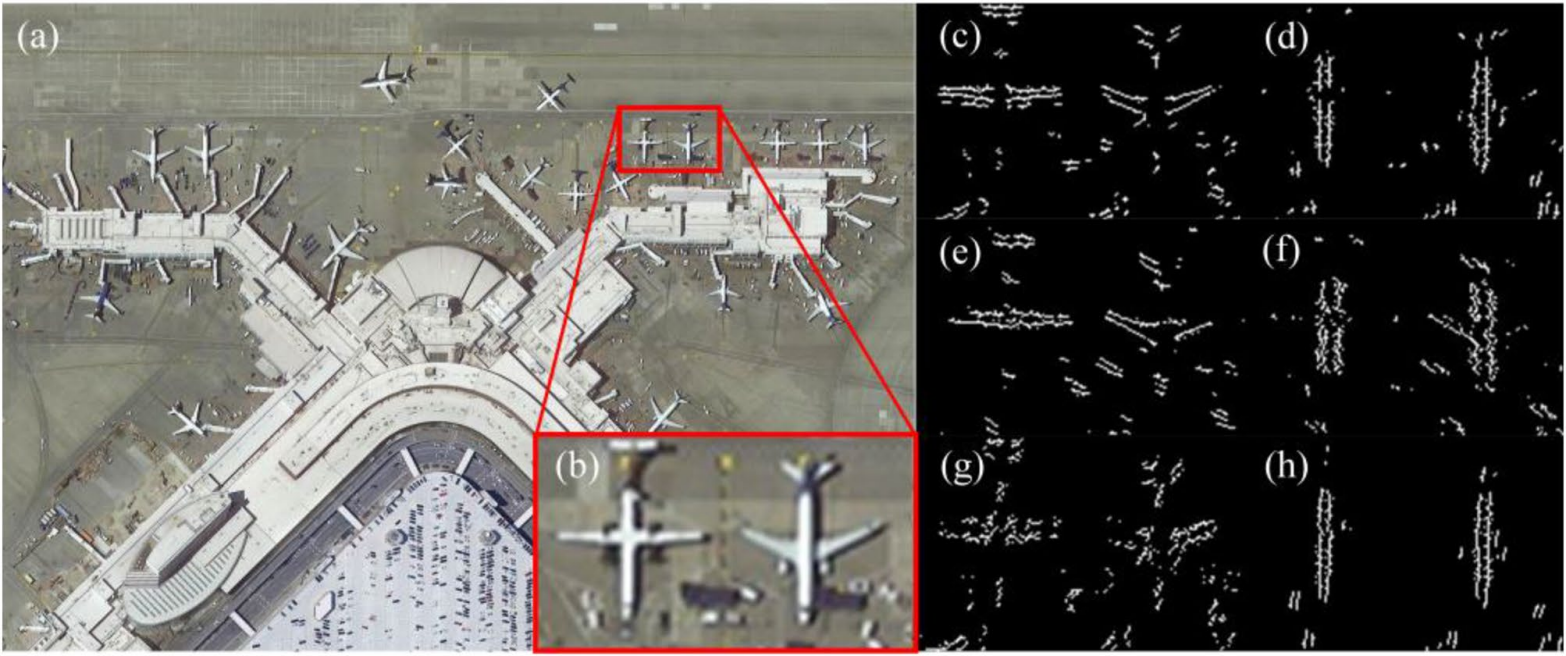
Edge images of the imaging experiment results when the length–width ratio of the rectangular pupil is 8. The ground resolution of the image is 0.4 m, signifying that every pixel corresponds to an approximate real-world ground distance of 0.4 m. The original remote sensing scene was acquired by the WorldView-3 satellite. (**a**) Original scene. (**b**) Local enlargement image. (**c**) Horizontal edge image of the original scene. (**d**) Vertical edge image of the original scene. (**e**) Horizontal edge image of the imaging experiment result taken when the rotation angle of the rectangular pupil is 0. (**f**) Vertical edge image of the imaging experiment result taken when the rotation angle of the rectangular pupil is 0. (**f**) Horizontal edge image of the imaging experiment result taken when the rotation angle of the rectangular pupil is 90. (**f**) Vertical edge image of the imaging experiment result taken when the rotation angle of the rectangular pupil is 90.

In our imaging experiment, we have selected remote sensing images containing various scenes, such as farmlands, airports, forests, residential areas, harbors, and sea surface, as the target scenes (as depicted in Fig. 5). These images were acquired by the WorldView-3 satellite and were downloaded from the official website of Maxar Technologies Inc. (https://www.maxar.com/). First, we present some imaging results for qualitative evaluation, as illustrated in Figs. 6, 7 and 8. To more clearly demonstrate the differences of degraded images in different directions and verify the “spatial asymmetry” characteristics of the RSA system, we have extracted and compared the horizontal and vertical edges of the original scene with the experimental images taken at different rotation angles of the rectangular pupil element, as depicted in Fig. 9.

Based on the above figures, the following observations can be made:

- The image acquired by the RSA system exhibits obvious non-uniform blur degradation and reduced resolution. Specifically, when the rectangle primary mirror possesses a large length–width ratio (as illustrated in Fig. 6, with a length–width ratio of 8), the image quality severely degrades, making it challenging to meet interpretation requirements.
- Geometric deviations exist between the degraded images at various moments during a rotation cycle of the rectangular primary mirror, i.e., at different rotation angles of the rectangular primary mirror (as depicted in Fig. 8).
- The resolution of degraded images varies significantly in different directions. Particularly, in the direction of the short side of the primary mirror, the image’s resolution is notably lower than that in the direction of the long side, which is evident in Fig. 9.

To quantify the influence of the RSA system on image degradation, we employed objective indices, namely PSNR and SSIM^24^, to quantitatively evaluate the degradation degree of the experimental images compared with the original scene.

The SSIM value ranges from 0 to 1, with a higher value indicating greater similarity between the two images. The formula for SSIM is defined as follows:

4 $${\text{SSIM}}\left( {H,D} \right) = \frac{{(2\mu_{d} \mu_{h} + c_{1} )(2\sigma_{dh} + c_{2} )}}{{(\mu_{d}^{2} + \mu_{h}^{2} + c_{1} )(\sigma_{d}^{2} + \sigma_{h}^{2} + c_{2} )}}$$

where *H* represents the original high-quality image, *D* represents the degraded image, $\mu_{d}$ and $\mu_{h}$ denote the means of the degraded image and the original high-quality image, respectively. $\sigma_{d}^{2}$ and $\sigma_{h}^{2}$ represent the variances of the degraded image and the original high-quality image, while $c_{1}$ and $c_{2}$ are constants utilized to ensure stability. $\sigma_{dh}$ represents the covariance between the two images.

PSNR is defined by:

5 $${\text{PSNR}}\left( {H,D} \right) = 10\lg \frac{{(2^{k} - 1)^{2} M_{1} M_{2} }}{{\sum\limits_{x = 1}^{{M_{1} }} {\sum\limits_{y = 1}^{{M_{2} }} {\left[ {H\left( {x,y} \right) - D\left( {x,y} \right)} \right]^{2} } } }}$$

where *H* denotes the original high-quality image, while *D* represents the degraded image. $M_{1}$ and $M_{2}$ correspond to the length and width of the image, respectively. Additionally, for 8-bit digital images, *k* is set as 8.

**Figure 10:**
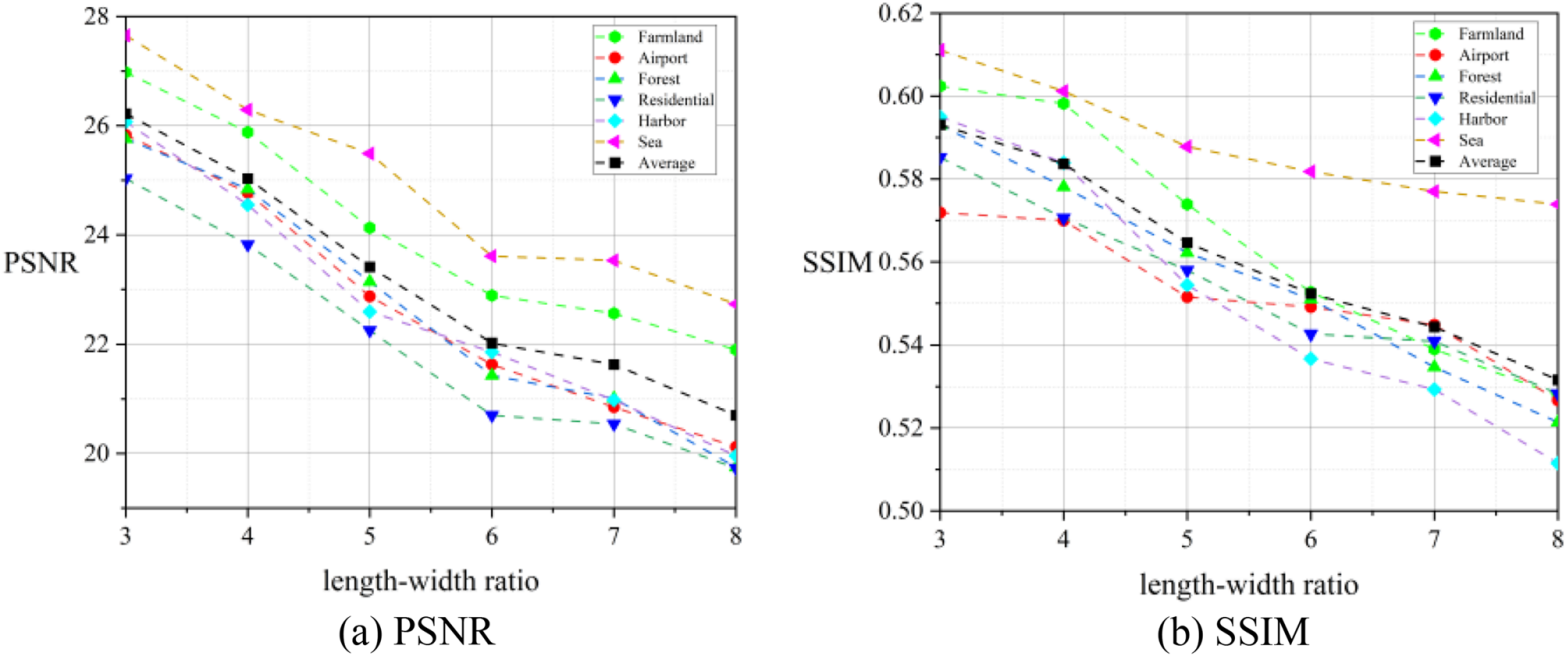
Evaluation results of image quality degradation.

Table 1 and Fig. 10 present the evaluation results of various target scenes under 6 different length–width ratios (averaged over 36 rotation angles from 0 to 180 in steps of 5 degrees).

**Table 1:** Evaluation results of image quality degradation. The unit of PSNR is decibel (dB).

Scene type	Length–width ratio 3		Length–width ratio 4		Length–width ratio 5		Length–width ratio 6		Length–width ratio 7		Length–width ratio 8		Average	
	PSNR	SSIM	PSNR	SSIM	PSNR	SSIM	PSNR	SSIM	PSNR	SSIM	PSNR	SSIM	PSNR	SSIM
Farmland	26.98	0.6024	25.88	0.5982	24.13	0.5739	22.89	0.5527	22.56	0.539	21.90	0.5278	24.06	0.5657
Airport	25.83	0.5719	24.77	0.5700	22.87	0.5515	21.63	0.5491	20.85	0.5448	20.12	0.5267	22.68	0.5523
Forest	25.76	0.5930	24.84	0.5781	23.14	0.5622	21.43	0.5511	21.01	0.5347	19.75	0.5214	22.66	0.5567
Residential	25.03	0.5852	23.82	0.5706	22.25	0.5582	20.70	0.5426	20.54	0.5409	19.73	0.5283	22.01	0.5543
Harbor	26.07	0.5954	24.55	0.5839	22.59	0.5544	21.86	0.5367	20.98	0.5293	19.96	0.5115	22.67	0.5518
Sea	27.65	0.6111	26.29	0.6012	25.49	0.5878	23.61	0.5818	23.53	0.5770	22.73	0.5739	24.93	0.5888
Average	26.22	0.5931	25.03	0.5837	23.41	0.5646	22.02	0.5523	21.63	0.5443	20.70	0.5316	23.17	0.5616

After further analysis of the quantitative experimental results, the following conclusions can be drawn:

- The degree of degradation of images obtained by the RSA system primarily depends on the length–width ratio of the rectangular primary mirror. According to the average results of each scene in Table 1, when the length–width ratio is small (no more than 4), the PSNR and SSIM remain above 25dB and 0.58, respectively. However, when the length–width ratio is large (no less than 7), the PSNR and SSIM drop below 22 dB and 0.55, respectively.
- The quality of images obtained by the RSA system deteriorates more significantly for scenes with rich texture information such as airports, harbors, residential areas, and forests. Compared to flat scenes like the sea surface and farmland, the PSNR and SSIM are approximately 10% and 5% lower, respectively.

**Figure 11:**
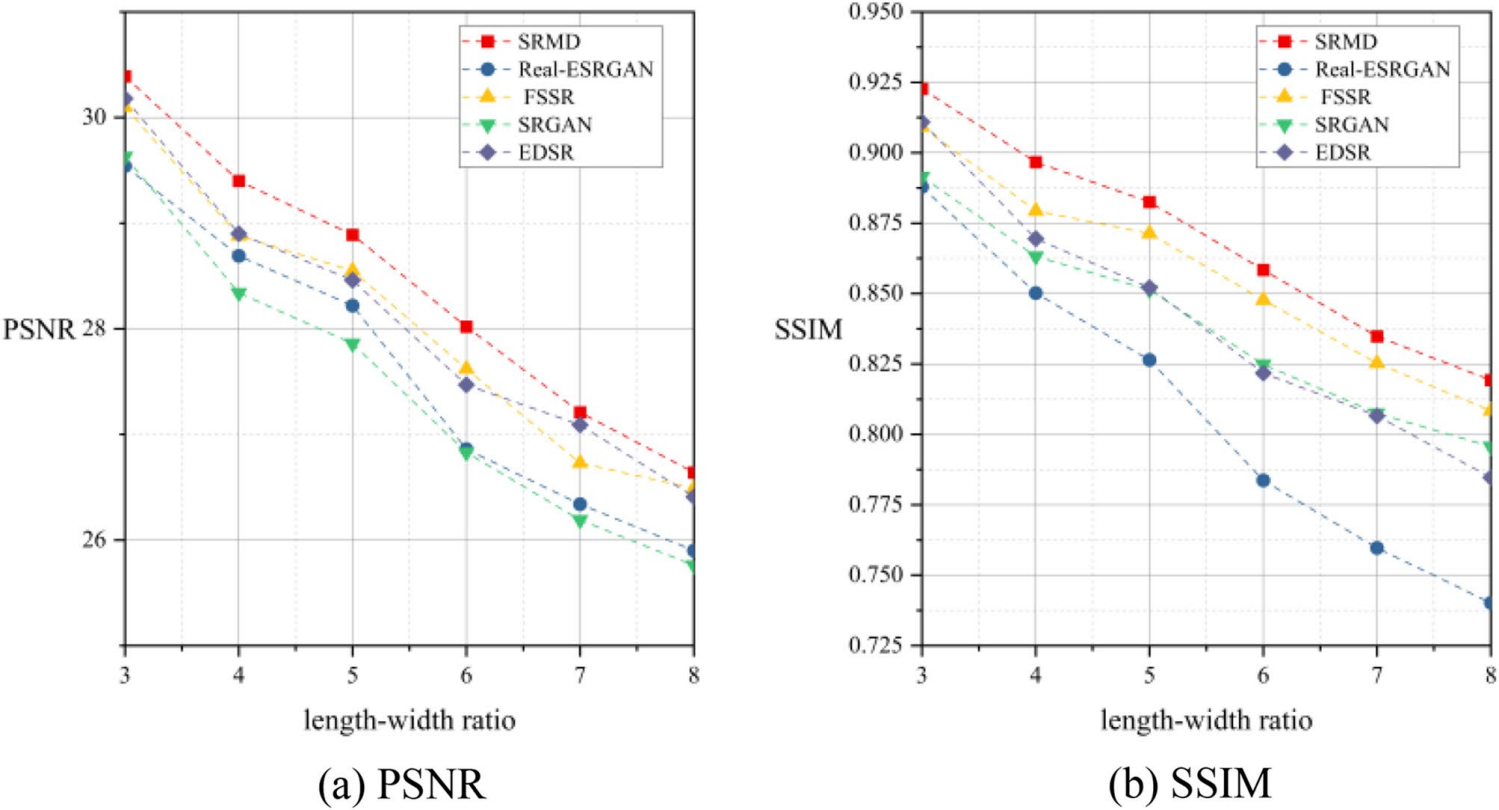
Results of image super-resolution (SR) experiment.

To further explore the unique imaging characteristics of the RSA system and the challenges it poses for enhancing image quality, as well as to provide a reference for research on corresponding image quality improvement methods, we utilized several representative deep learning-based approaches, namely SRGAN, EDSR, SRMD, FSSR, and Real-ESRGAN, to conduct image SR experiments for the RSA system. Table 2 and Fig. 11 depict the average results for all test images. Based on the quantitative evaluation results in the table, we observed that SRMD performs slightly better than the other methods. Comparing Tables 1 and 2, when the length–width ratio is 3, SRMD’s results achieve a PSNR of 30.39 dB and an SSIM of 0.9227, respectively. This represents a 4.17 dB improvement in PSNR and a 0.3296 improvement in SSIM compared to the degraded images. As the length–width ratio increases, based solely on the two objective indices, it seems that all methods are still able to achieve commendable performance. When the length–width ratio is 8, the SRMD method stands out by achieving a remarkable 5.94 dB increase in PSNR and a 0.2877 improvement in SSIM. Meanwhile, SRGAN, despite performing the poorest in terms of the PSNR metric, manages to achieve a significant 5.06 dB improvement, while Real-ESRGAN, performing the worst according to the SSIM metric, achieves a 0.2086 improvement. However, it should be noted that there is a considerable degradation in image quality at this point, which results in the PSNR of the SR outputs remaining below 27 dB, while the SSIM remains below 0.82.

**Table 2:** Average super-resolution results for all test images. The unit of PSNR is decibel (dB).

Method	Length–width ratio 3		Length–width ratio 4		Length–width ratio 5		Length–width ratio 6		Length–width ratio 7		Length–width ratio 8	
	PSNR	SSIM	PSNR	SSIM	PSNR	SSIM	PSNR	SSIM	PSNR	SSIM	PSNR	SSIM
SRGAN	29.63	0.8915	28.34	0.8632	27.86	0.8513	26.83	0.8249	26.19	0.8075	25.76	0.7957
EDSR	30.18	0.9109	28.90	0.8695	28.46	0.8521	27.47	0.8218	27.09	0.8066	26.41	0.7847
SRMD	30.39	0.9227	29.40	0.8967	28.89	0.8825	28.02	0.8584	27.21	0.8348	26.64	0.8193
FSSR	30.10	0.9092	28.88	0.8794	28.55	0.8713	27.62	0.8477	26.73	0.8253	26.49	0.8084
Real-ESRGAN	29.54	0.8879	28.69	0.8502	28.22	0.8264	26.86	0.7837	26.34	0.7597	25.90	0.7402

**Figure 12:**
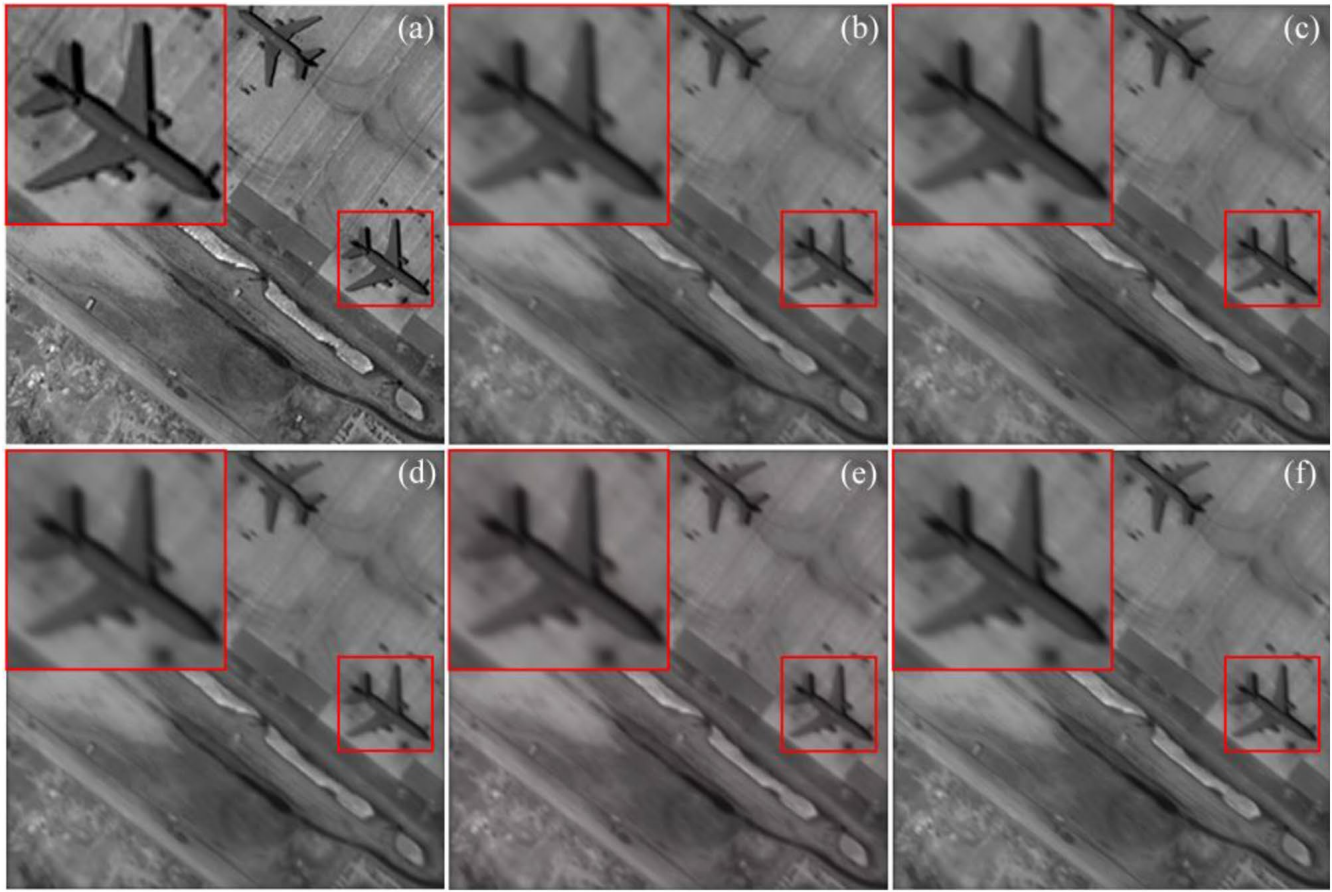
Original scene and SR results with the rotation angle 135° and the length–width ratio 6. The ground resolution of the image is 0.4 m, signifying that every pixel corresponds to an approximate real-world ground distance of 0.4 m. The original remote sensing scene was acquired by the WorldView-3 satellite. (**a**) Original scene; (**b**) SRGAN; (**c**) EDSR; (**d**) FSSR; (**e**) Real-ESRGAN; (**g**) SRMD.

**Figure 13:**
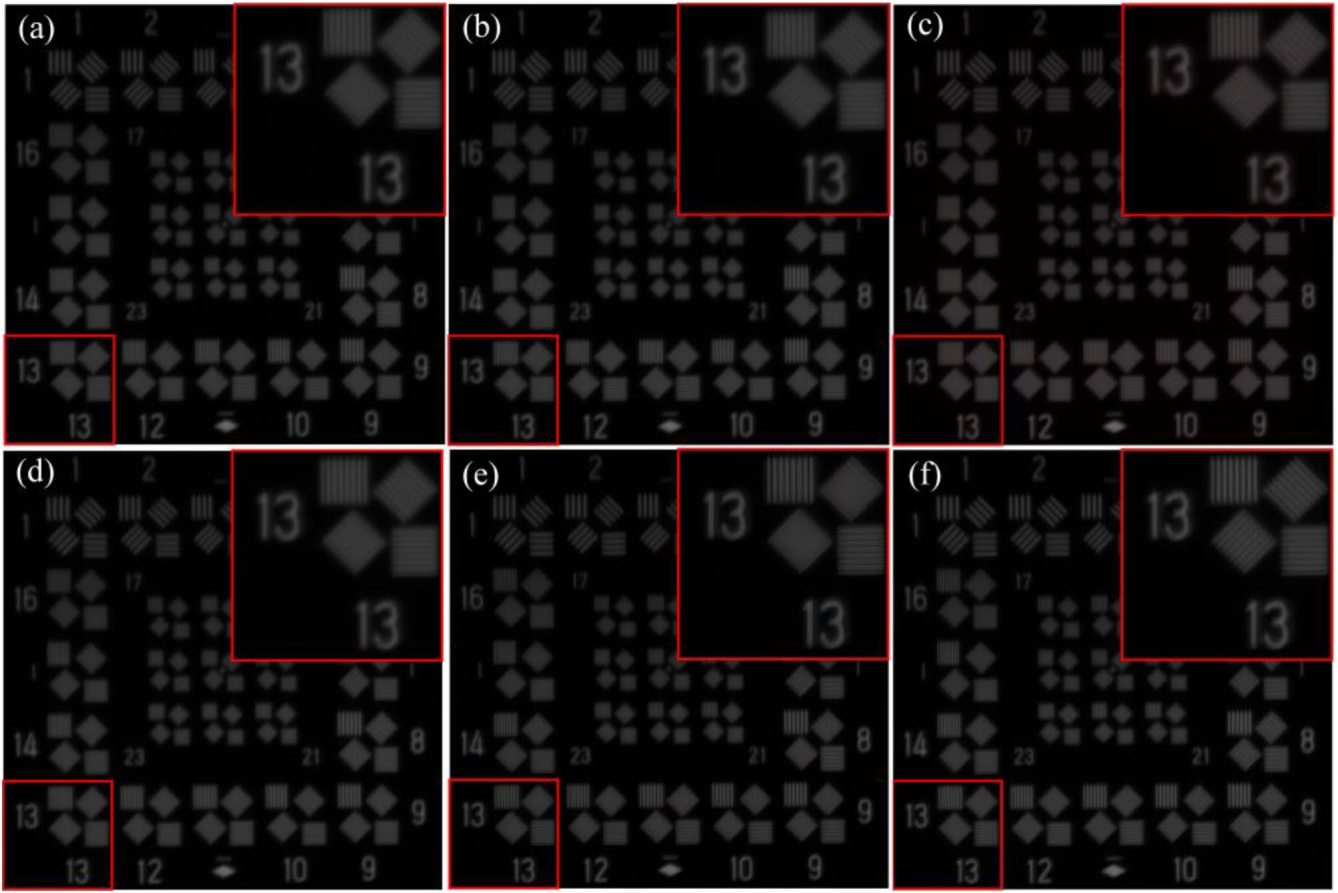
Degraded image and SR results of the resolution target image with the rotation angle 0° and the length–width 3. (**a**) Degraded image; (**b**) SRGAN; (**c**) EDSR; (**d**) FSSR; (**e**) Real-ESRGAN; (**f**)SRMD.

In addition to quantitative evaluations, visual results are provided to qualitatively assess the tested SR methods. As shown in Figs. 12 and 13, the issue of non-uniform resolution in the images has not been adequately addressed, resulting in a noticeable deterioration in image quality along the shorter side. Despite the possibility of partial detail recovery in directions with lower resolution in the image, the SR outputs may still fail to meet the requirements of interpretive applications, such as resolution targets other than the vertical direction depicted in Fig. 13. This indicates that while directly applying existing SR methods to the RSA system may yield some improvement in PSNR and SSIM, it is still inappropriate, especially when dealing with cases where the aspect ratio of the pupil is relatively large. We believe that the reason is the substantial reduction in image quality in the short side direction of the primary mirror, resulting in a lack of information for all the SR methods to produce satisfactory results. Therefore, targeted research is necessary to develop image SR methods based on the RSA system’s special characteristics. For instance, it is important to explore how to leverage the high-resolution information of the image itself to compensate for the significant information loss in the short side direction of the primary mirror.

## Conclusion

To investigate the imaging characteristics of the RSA system, we establish a dynamic imaging model based on theoretical analysis of the system’s imaging mechanism. An experimental platform is then constructed to simulate the blurring process of dynamic imaging in the RSA system. This platform utilizes a removable and rotary rectangular entry pupil optical component and its supporting structure to simulate the rotating imaging process of the rectangular primary mirror. To conduct imaging experiments, remote sensing images of various scenes, such as airports, harbors, residential areas, sea surface, forests, and farmlands, are used as target scenes. Through both subjective evaluations and objective measures, we analyze the effects of the rectangular primary mirror with different length–width ratios of the RSA system on the degradation of image quality. By comparing the imaging experiment results with theoretical analysis, this paper verifies the "spatially asymmetric and temporally periodic" imaging characteristics of the RSA system. Furthermore, we conduct image super-resolution experiments, evaluating the performance of several representative deep learning-based single image super-resolution methods and analyzing their limitations when directly applied to the images captured by the RSA system. This experimental work offers a reference for the development of the RSA technology. In future studies, we intend to explore and design image quality improvement methods specifically tailored for the RSA system, aiming to address the issue of non-uniform resolution in images.

## Data Availability

The datasets used during the current study available from the corresponding author on reasonable request.
